# Increased inflammation with crude *E. coli* LPS protects against acute leptospirosis in hamsters

**DOI:** 10.1080/22221751.2019.1710435

**Published:** 2020-01-09

**Authors:** Wenlong Zhang, Xufeng Xie, Jiaqi Wang, Ning Song, Tianbao Lv, Dianjun Wu, Naisheng Zhang, Yongguo Cao

**Affiliations:** a Department of Clinical Veterinary Medicine, College of Veterinary Medicine, Jilin University, Changchun, People’s Republic of China; b Key Laboratory for Zoonosis Research, Ministry of Education, College of Veterinary Medicine, Jilin University, Changchun, People’s Republic of China

**Keywords:** Leptospirosis, inflammation, treatment, LPS, hamster

## Abstract

Leptospirosis is a worldwide zoonotic disease that causes acute kidney injury, liver disease, bleeding disorders, and even death. Treatment of the disease is largely dependent on the use of antibiotics, but recent studies on pathogenesis of leptospirosis have shown that immunomodulation may also be an effective treatment for this disease. Since the delay in inflammation correlates with higher pathogenicity of leptospira, we studied the effect of inducing inflammation on leptospirosis by using TLR4 activator LPS. In accordance with our hypothesis, treatment with LPS protected against leptospirosis by enhancing the inflammatory response in hamsters. The gene expression levels of TLR2, TLR4, NLRP3 and inflammatory factors were higher in LPS-treated group during leptospira infection in hamsters. Although the levels of NO and iNOS were higher in LPS-treated group than in Leptospira-infected group, the protective effect induced by LPS is iNOS-independent. Treatment with LPS induced higher anti-leptospira IgG level than infection with leptospira alone. Then, expressions of costimulatory molecules and maturation markers were analysed. The data showed that treatment with LPS enhanced the expression of CD40, CD80 and CD86. Our results indicate that increased inflammation induced by LPS derived from *Escherichia coli* (*E. coli*) protects against leptospirosis in hamsters.

## Introduction

Leptospirosis, caused by pathogenic leptospira (Lep), is a globally spread zoonotic disease [1]. Humans and animals contract leptospirosis by direct contact with infected animals or by ingesting soil or water contaminated with leptospira [2]. Infection through skin abrasion or sodden skin is also very frequent. Infected hosts present a diverse array of clinical manifestations ranging from asymptomatic forms to jaundice, renal failure, bleeding disorders and even death [3]. Although new antimicrobial drugs are being evaluated, antibiotics, such as doxycycline and penicillin, are currently the most widely used therapeutic against leptospirosis [4,5]. Previous research reported that Toll-like receptor (TLR) 2 agonist Pam3CSK4 alleviates the pathology of leptospirosis in hamsters but only has a limited effect on survival rate [6]. The efficacy of additional immune activators against leptospirosis has not been investigated.

Leptospira-induced gene expression profiles are different between susceptible hamsters and resistant mice. During leptospira infection, the induction of proinflammatory mediators is delayed in hamsters compared with mice [7]. Thus the early inflammatory response is important to resist leptospirosis. That is why some immunosuppressive drugs, such as thalidomide and cyclosporine, fail to cure leptospirosis [8,9]. TLRs acting as pattern recognition receptors (PRRs) can recognize a variety of pathogen-associated molecular patterns (PAMPs) [10]. In particular, TLR2 and TLR4 are vital for the recognition of leptospira and control of the leptospiral burden *in vivo* [11,12]. In consideration of the modest efficacy of TLR2 agonist Pam3CSK4 against leptospirosis, we hypothesized that TLR4 agonist lipopolysaccharide (LPS) could provide better protection.

Since inflammation is an important host defense against leptospirosis at the early stage, strengthening the inflammatory response may be beneficial for the overall survival of hamsters. LPS derived from *E. coli* is a well-characterized inducer of inflammatory response *in vivo* [13,14] that activates cytokine expression via NFκB and MAPK signalling pathway in a TLR4-dependent manner [15]. LPS from leptospira is less virulent than that of *E. coli*. TLR2/TLR1 are the predominant receptors to leptospiral LPS in human cells, whereas TLR2 but also TLR4 contributed to activation in murine cells [16].

In this study, we determined whether LPS from *E. coli* contributes to defense against leptospirosis and found that LPS plays a protective role by augmenting inflammation and decreasing bacterial burden in hamsters. Our results point to the possibility of treating leptospirosis by increasing inflammation level.

## Materials and methods

### Ethics statement

Hamsters were maintained on standard rodent chow with water supplied ad libitum and with a 12-h light/12-h dark cycle during the experimental period. All animal experiments were performed according to regulations of the Administration of Affairs Concerning Experimental Animals in China. The protocol was approved by the Institutional Animal Care and Use Committee of Jilin University (20170318).

### Bacterial strains and animals

Pathogenic *Leptospira interrogans* serovar Lai strain Lai (56601) was used to infect hamsters. Leptospira was grown in liquid Ellinghausen-McCullough-Johnson-Harris (EMJH) medium at 29°C. The virulence of the leptospira was maintained by passage in hamsters. Leptospira was passaged *in vitro* less than three times in liquid EMJH for all infection studies. Before infection, the concentration of leptospira was determined using a Petroff-Hausser counting chamber and a dark-field microscope. Syrian golden hamsters (*Mesocricetus auratus*) were provided by the Liaoning changsheng biotechnology co. LTD.

### Effect of LPS on leptospiral growth

To analyse the influence of LPS on leptospiral growth, 10^7^ leptospires in 0.5 ml of EMJH medium supplemented with 0.5 ml of LPS (0, 10 μg/ml) (Sigma, USA), which was dissolved in DMEM medium, was cultured at 37°C for 1 h. The LPS activity that we used was 6.8 × 10^5^ EU/mg by End-point Chromogenic Tachypleus Amebocyte Lysate assay (Xiamen Bioendo Technology Co., China). This process mimicked what happens to leptospira with LPS in the body. Then 30 μl cultures were recultured in 3 ml EMJH at 29°C. Growth was analysed for 4 days by using a Petroff-Hausser chamber and a dark-field microscope.

### Experimental infections

Four- to six-week-old female hamsters were injected intraperitoneally with LPS (10 μg/100 g) (*n *= 4) or vehicle (*n *= 4) 24 h before intraperitoneal inoculation with 10^7^ leptospira. Then, LPS or vehicle injection was performed once daily on days 0–3 (infection was on day 0) post-infection (p.i.), and animals were observed no less than three times daily for a period of 21 days after infection [17]. Then the survival rate of hamsters was studied. The solvent for LPS was DMEM medium. Vehicle was the solvent for LPS. The animal experiment was repeated three times.

To detect the effect of LPS on inflammation levels of hamsters in leptospirosis, the animal infection experiments were repeated. At each time point, three hamsters of each group were humanely euthanized by using CO_2_ and organs (liver, kidney, lung and spleen) of hamsters were collected. Then, the histology, myeloperoxidase (MPO) activity, leptospira burdens and analyses of gene expressions were conducted in target organs of hamsters.

To explore the role of iNOS on LPS-induced protective effect and the cytotoxicity of LPS on hamsters, hamsters were divided into three groups (*n *= 8). Hamsters received 4-aminopyridine (AP) and/or LPS intraperitoneally a day prior to Leptospira infection or LPS injection with consecutive 4 days corresponding drugs injection. Then the survival rate of hamsters was studied. Moreover, hamsters were injected intraperitoneally with LPS (*n *= 4) or vehicle (*n *= 4) for 5 days. Then the weight of hamsters was recorded.

### Histopathological examination

At the time of necropsy, the primary organs (liver, kidney and lung) of hamsters were removed immediately and fixed in 10% neutral buffered formalin; dehydrated, paraffin-embedded, and sliced, followed by staining with hematoxylin and eosin (H&E). Pathological changes were examined and graded as described previously by using a microscope (Olympus, Japan) [18].

### Myeloperoxidases (MPO) activity examination

The MPO activity in kidney, liver and lung of hamsters was evaluated using test kits purchased from Nanjing Jiancheng Bioengineering Institute (China) according to the manufacturer's protocols.

### Bacterial load and PCR assay

The leptospiral burdens in organs of hamsters were determined by quantitative PCR (qPCR). The DNA extraction was followed as previously described [17]. The DNA concentration was measured by spectrometry. The primers used were specific for the lipL32 gene as described previously [19]. The qPCR reaction was performed using an Applied Bioscience 7500 thermocycler and FastStart Universal SYBR Green Master (Roche Applied Science, Mannheim, Germany). The number of leptospira was measured using a standard curve prepared from serial dilutions (10^9^–10^2^) of genomic DNA extracted from in vitro-cultivated bacteria and the results were presented as the number of genome equivalents per mg of tissue DNA.

### RT-qPCR

Total RNA of organs was extracted using TRIzol (Invitrogen, USA) following the manufacturer's instructions. RNA was reverse transcribed into cDNA by using random primers from a TransScript One-Step gDNA Removal kit and cDNA Synthesis SuperMix (TransGen Biotech, China). The primers used in this study were listed in Table 1. The qPCR reaction was performed as previous study [20]. The number of target gene was normalized to GAPDH using a 2^−ΔΔCT^ method.

**Table 1.** Sequence of primers used for qPCR assays

Gene	Primer	Sequence (5′-3′)
Hamster GAPDH	Sense	GATGCTGGTGCCGAGTATGT
	Anti-sense	GCCACGCCCACATCATTC
Hamster TLR2	Sense	TGTTTCCCGTGTTACTGGTCAT
	Anti-sense	CACCTGCTTCCAGACTCACC
Hamster TLR4	Sense	ACGACGAGGACTGGGTGAGA
	Anti-sense	GCCTTCCTGGATGATGTTGG
Hamster NLRP3	Sense	TGAATCTGGGCAACAACGAC
	Anti-sense	CCAAGAAGGCTCAAAGACAAC
Hamster IL-10	Sense	AAGGGTTACTTGGGTTGCC
	Anti-sense	AATGCTCCTTGATTTCTGGC
Hamster TNF-α	Sense	GGTGATACCAGCAGACGG
	Anti-sense	CTTGATGGCGGACAGGA
Hamster IL-1β	Sense	TTCTGTGACTCCTGGGATGGT
	Anti-sense	GTTGGTTTATGTTCTGTCCGTTG
Hamster iNOS	Sense	GGAGCGAGTTGTGGATTGTC
	Anti-sense	CCTGGGAGGAACTGATGGA
Hamster CD40	Sense	GCCCTGGCTTTGGAGTTA
	Anti-sense	AGACAGCGTCGGTCGTATT
Hamster CD80	Sense	TCTCTTTGTGCTGCTGGTTG
	Anti-sense	CCAGTAGATTCGGAGTATGTTTAG
Hamster CD86	Sense	GCCCATTTACAAAGGCTCAA
	Anti-sense	GCTCCGTATCTGTCTGCTGG
Hamster MHCII	Sense	CCTGAGGTGACCGTGTTCC
	Anti-sense	ACCGTCTGTGACTGGCTTG

### NO examination

Kidney, liver and lung of hamsters were homogenized with Cell and Tissue Lysis Buffer for Nitric Oxide Assay (Beyotime, China). Then the level of NO was measured using test kits purchased from Beyotime Biotechnology (China) according to the manufacturer's protocols.

### Western blot analysis

Proteins of leptospira were extracted as previously described [21]. Protein samples (30 μg/well) were separated and transferred onto the polyvinylidene difluoride membrane. After blocking, the membrane was incubated with primary antibodies and secondary antibodies. Then membranes were tested by ECL Plus Western Blotting Detection System (Amersham Life Science, UK). The primary antibodies used were antiserum (1:20) of hamsters from untreated group, LPS group, Lep group and Lep+LPS group. The secondary antibodies used were HRP-conjugated goat anti-hamster IgG (Abcam USA).

### Anti-leptospira ELISA

Leptospira was collected by centrifuging at 12,000 g for 5 min, after which leptospira was resuspended in PBS and coated overnight at 4°C in 96-wells plates at a density of 10^7^. Then plates were blocked for 1 h at room temperature with PBS-5% BSA, washed, and the doubled serum dilutions of hamsters were added to the plates for 2 h at room temperature. After washing, HRP-conjugated goat anti-hamster IgG was added for1 h at room temperature. The plates were washed and peroxidase activity was revealed by TMB substrate (Sigma, USA), and stopped by HCL 1N. Reading was performed at 450 nm. The titres of the sera were expressed as the dilution corresponding to twice the background level of the ELISA [11]. Alternatively, same dilutions of the different sera were also compared at 450 nm.

### Data analysis

Survival differences between the study groups were compared by using the Kaplan-Meier log-rank test. Comparisons between groups were performed by using *t*-test. Differences were considered significant at *P* < 0.05.

## Results

### Treatment with LPS protects against leptospirosis by raising inflammation levels in hamsters

**Figure 1.**
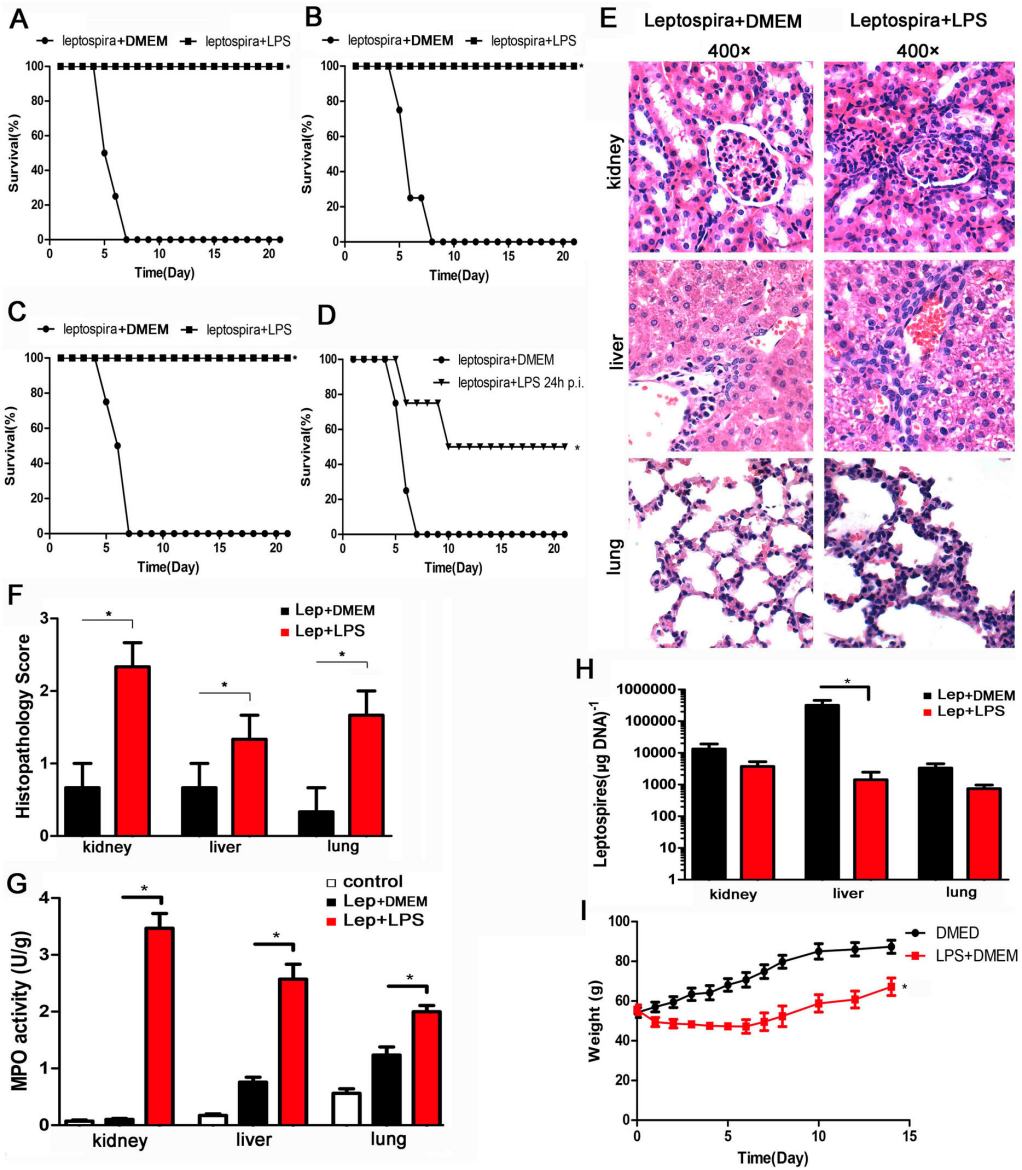
Treatment with LPS protects against leptospirosis with the improving inflammation levels in hamsters. (A–C) The animal experiment was repeated three times. Survival curves of hamsters in the infected control group (*n *= 4) and the group treatment with LPS (*n *= 4). *P < 0.05 versus infected controls as determined by a Kaplan-Meier log rank test. (D) A single dose of LPS also contributed to the survival of hamsters. Survival differences between study groups (*n *= 4) were compared using the log-rank test. *P < 0.05 vs. the Lep group. (E) Histopathology of kidneys, livers, and lungs of hamsters in the infected control group and the LPS-treated group. Magnification, ×400. Samples were collected at 2 d p.i., and representative photographs are presented. (F) Histopathology scores for kidneys, livers, and lungs of hamsters. The data represent the mean histopathology scores for the two groups of hamsters. Statistical analysis of the results for infected controls (*n *= 3) and the LPS-treated group (*n *= 3) was performed by using the Wilcoxon rank sum test. *P < 0.05. (G) MPO activity in kidneys, livers and lungs of hamsters in infected controls and LPS-treated group at 2 d p.i.. Data are presented as mean ± SEM and analysed by *t*-test. *P < 0.05. (H) Leptospiral burdens in the kidneys, livers, and lungs of hamsters in the infected control group (*n *= 3) and the LPS-treated group (*n *= 3) was determined by qPCR. Samples were collected at 4 d p.i.. The results are presented as numbers of genome equivalents per microgram of tissue DNA, and the differences were compared by *t*-test. *P < 0.05. (I) Weight changes in control group (*n *= 4) and LPS-treated group (*n *= 4). The differences were compared by *t*-test. *P < 0.05.

After leptospira infection, all hamsters in the untreated group died. The animal experiment was repeated three times. Treatment with LPS improved the survival rate of hamsters to 100 percent (Figure 1(A–C)). A single dose of LPS also contributed to the survival of hamsters (Figure 1(D)). LPS had no effect on leptospiral growth *in vitro*, ruling out bactericidal effect (data not shown). Then the animal infection experiments were repeated and the samples were collected at 2day and 4 day p.i.. Representative photographs of hamster kidneys, livers and lungs were selected from the infected controls and LPS-treated group at 2 day p.i. (Figure 1(E)). Treatment with LPS aggravated the pathological changes of kidney, liver and lung with more inflammatory cell infiltration and higher lesion grade than infected controls (Figure 1(E,F)). Furthermore, the myeloperoxidase (MPO) activity, a biological readout for the degree of inflammation, was higher in LPS-treated group compared with infected controls (Figure 1(G)). Leptospira burden of kidney, liver and lung was comparable between infected controls and LPS-treated group at 2 day p.i. (data not shown). However, leptospira burden of liver in LPS-treated group was significantly lower than that of infected controls at 4 day p.i. (Figure 1(H)). Although there were no deaths after treatment with LPS for 5 days, the weight gain of the LPS-treated group was slower than that of control group (Figure 1(I) and Figure 3(C)). These results indicated that treatment of hamsters with LPS protected against leptospirosis by further inducing inflammation.

### Treatment with LPS elevates gene expression of TLR2, TLR4, NLRP3 and inflammatory factors during leptospira infection in hamsters

**Figure 2.**
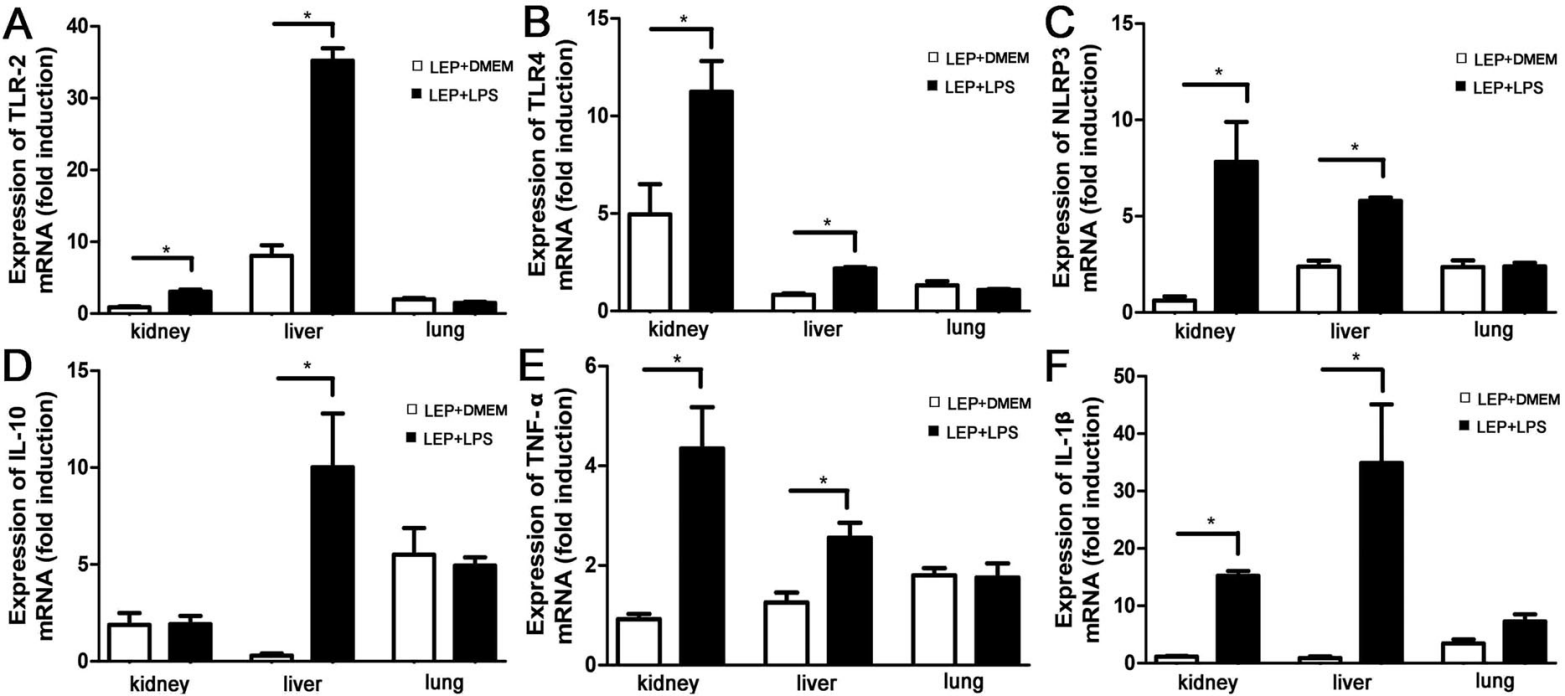
Gene expressions of TLR2, TLR4, NLRP3 and cytokines in tissues after injection of leptospires. Samples were collected from infected controls (*n *= 3) and LPS-treated group (*n *= 3) at 2 d p.i.. TLR2 (A), TLR4 (B), NLRP3 (C), IL-10 (D), TNF-α (E) and IL-1β (F) mRNA levels were quantified by RT-qPCR. The results were normalized to the expression level of the housekeeping gene GAPDH. The levels of these genes in different tissues from three healthy individuals were given a value of 1.0. Different mRNA expression levels between the infected controls and LPS-treated group were compared by *t*-test. *P < 0.05.

The early induction of TLR2, TLR4 and inflammatory factor is important for controlling leptospirosis [6,7]. To study the influence of LPS on leptospira-induced early inflammatory response, samples were collected and used for RT-qPCR at 2 day p.i.. The expression levels of TLR2, TLR4 and NLRP3 were higher in LPS-treated group than infected controls (Figure 2(A–C)). Expression of both pro-inflammatory factors, TNF-α and IL-1β, and anti-inflammatory IL-10 were elevated after treatment with LPS compared with infected controls (Figure 2(D–F)).

### Treatment with LPS increases the levels of NO and iNOS during leptospira infection in hamsters

**Figure 3.**
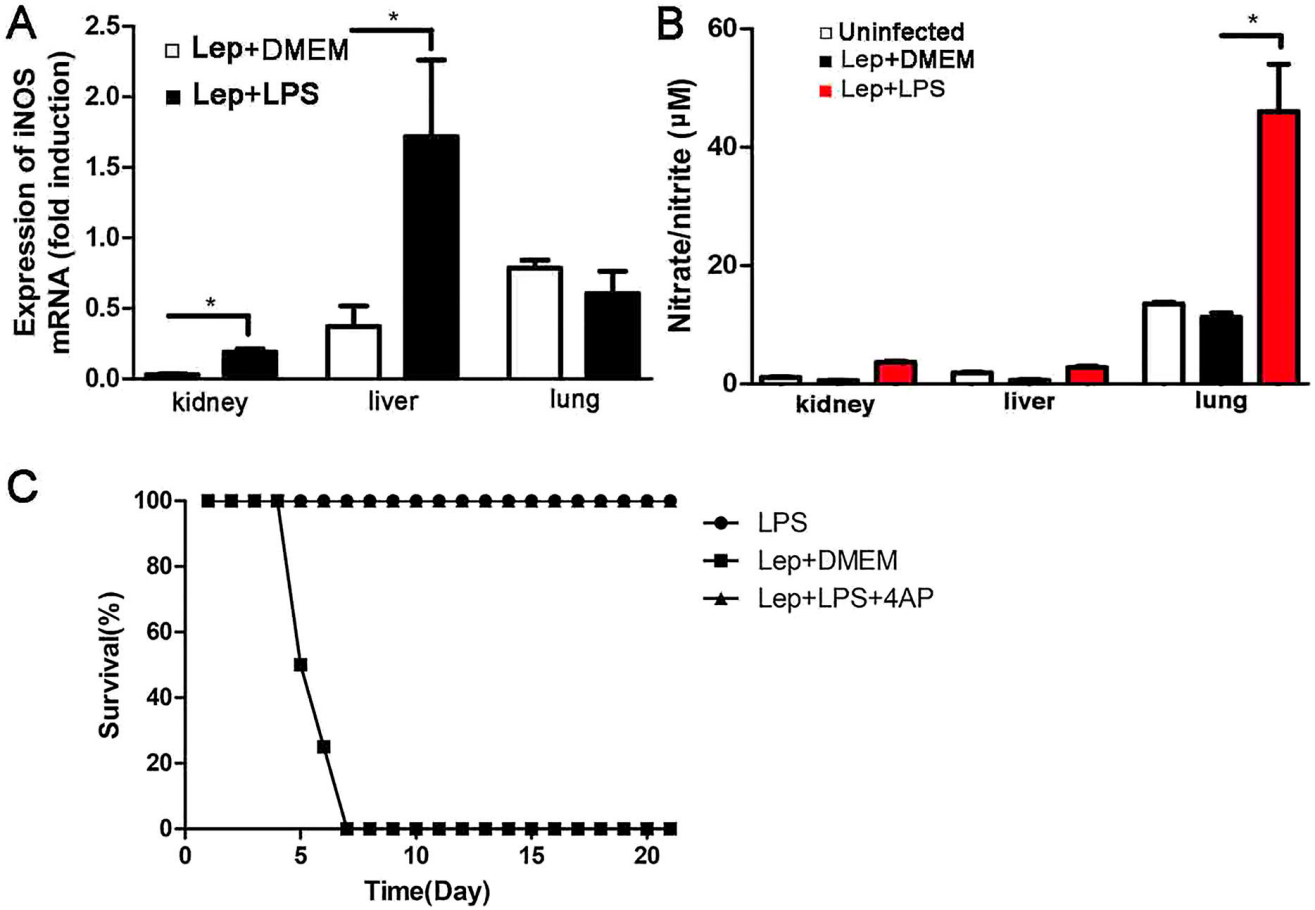
Treatment with LPS increases the levels of NO and iNOS during leptospira infection in hamsters. (A) Gene expression of iNOS in organs was detected by RT-qPCR at 2 d p.i.. The results were normalized to the expression level of the housekeeping gene GAPDH. The levels of iNOS in different tissues from three healthy individuals were given a value of 1.0. Different mRNA expression levels between the infected controls and LPS-treated group were compared by *t*-test. *P < 0.05. (B) The expression of NO was quantified by detecting the concentration of nitrate/nitrite. Samples were collected at 2 d p.i. from different groups. Data are presented as mean ± SEM. *P < 0.05 versus infected controls as determined by a *t*-test. (C) The protective effect induced by LPS is iNOS-independent and the dosage of LPS on hamster is suitable. Survival differences between study groups (*n *= 8) were compared using the log-rank test. *P < 0.05 vs. the Lep group.

Previous research shows that the expression of inducible nitric oxide synthase (iNOS) and the resulting nitric oxide (NO) have an important role against leptospirosis [22]. In this study, the levels of NO and iNOS in kidney, liver and lung were detected at 2 day p.i.. Treatment with LPS increased iNOS expression compared with infected controls by using RT-qPCR (Figure 3(A)), and the level of NO in the LPS-treated group was also higher than in infected controls, especially in the lung (Figure 3(B)). Co-treatment of LPS with iNOS-inhibitor 4AP did not reduce the survival of leptospira-infected hamsters (Figure 3(C)), indicating that the protective effect induced by LPS is iNOS-independent.

### Treatment with LPS improves the anti-leptospira IgG level

**Figure 4.**
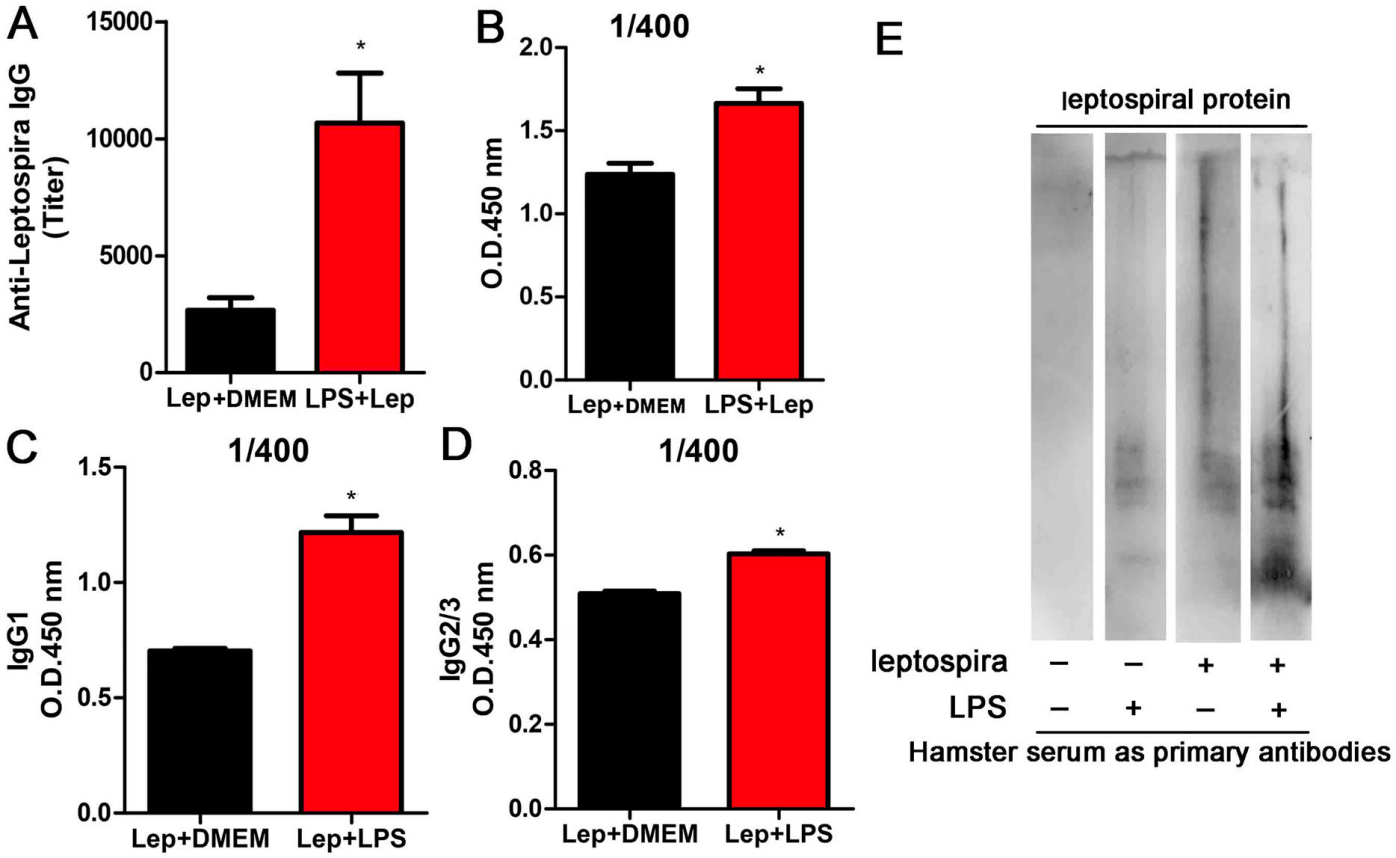
Treatment with LPS improves the anti-leptospira IgG level. The serum and spleens of hamsters in different groups were collected at 4 d p.i.. (A) Titres of anti-leptospira IgG was measured in the serum of different groups and analysed by the t-test. *P < 0.05. (B) Anti-Leptospira IgG quantification in sera from infected controls and LPS-treated group. Data are expressed as the OD at 450 nm measured in sera (*n *= 3 per group) at the dilution 1/400, and the differences were compared by *t*-test. *, *P *< 0.05. (C–D) Anti-Leptospira IgG1 and IgG2/3 quantification in sera from infected controls and LPS-treated group. Data are expressed as the OD at 450 nm measured in sera (*n *= 3 per group) at the dilution 1/400, and the differences were compared by *t*-test. *P < 0.05. (E) Western blot about anti-Leptospira IgG from different groups.

Since treatment with LPS strengthened the innate immune response during leptospirosis, we reasoned that the anti-leptospira antibody level might also be enhanced. To verify our conjecture, anti-leptospira IgG level in serum of hamster was measured at 4 day p.i. by anti-leptospira ELISA. As expected, treatment with LPS improved the titre of antisera compared with infected controls accompanied by elevated subtypes (IgG1 and IgG2/IgG3) (Figure 4(A,C,D)). Different sera were diluted 400 times and measured at 450 nm. The LPS-treated group showed higher light absorption than infected controls (Figure 4(B)), and the reactivity of serum from LPS-treated infection group against Leptospiral proteins displayed greater complexity compared with serum from other groups (Figure 4(E)). These results indicated that treatment with LPS improved the anti-leptospira IgG level.

### Treatment with LPS enhances expressions of maturation markers in spleen

**Figure 5.**
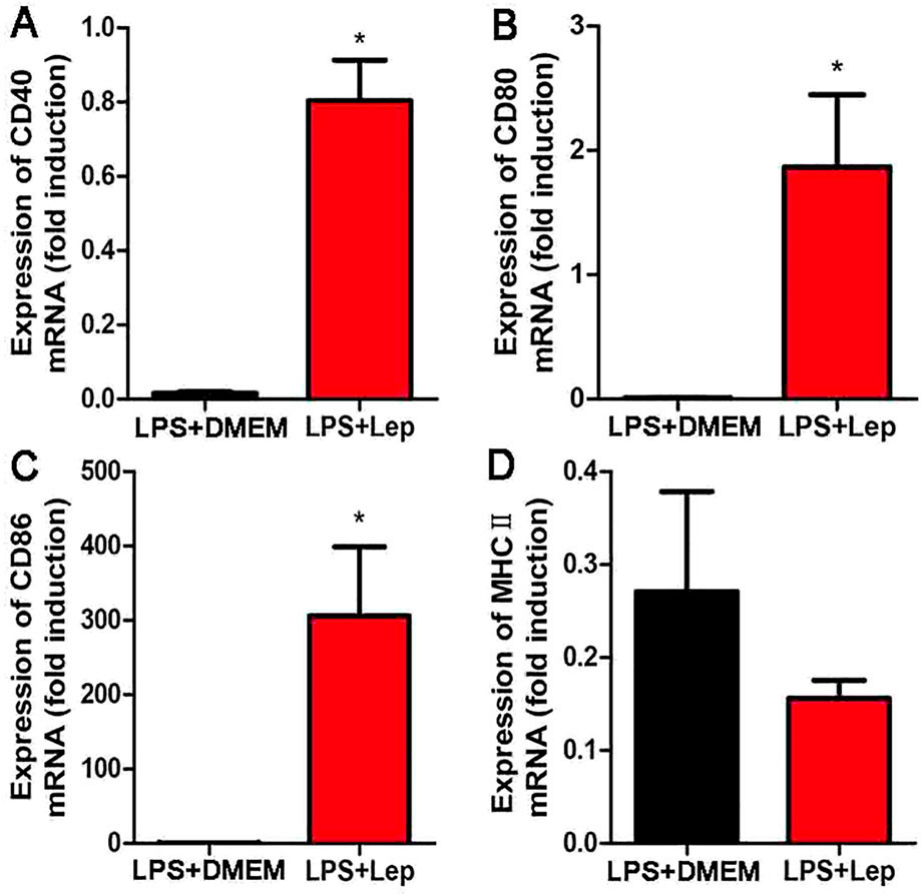
Treatment with LPS improves the expressions of maturation markers. (A–D) Gene expressions of CD40, CD80, CD86 and MHCII in organs were detected by RT-qPCR. The results were normalized to the expression level of the housekeeping gene GAPDH. The levels of these genes in different tissues from three healthy individuals were given a value of 1.0. Different mRNA expression levels between the infected controls and LPS-treated group were compared by *t*-test. *P < 0.05.

To study the influence of LPS on acquired immunity, the expressions of costimulatory molecules and maturation markers were detected at 4-day p.i. by RT-qPCR in spleen. Treatment with LPS enhanced the expressions of CD40, CD80 and CD86 compared with infected controls (Figure 5(A–C)). The level of costimulatory molecules MHCⅡwas lower in the LPS-treated group than in infected controls, but the difference was not significant (Figure 5(D)). These results indicated that treatment with LPS improved acquired immunity and enhanced expression of maturation markers in spleen.

## Discussion

Leptospirosis, a globally spread zoonotic disease, induces a variety of symptoms, which range from a mild illness to severe infection and even death [23]. Because of non-specific presentation and poor diagnostics [24], the mainstay of treatment is empiric antibiotic therapy with a broad-spectrum agent. Besides antibiotics, effective immunomodulators have not been reported. Our previous research showed the efficacy of TLR2 agonist Pam3CSK4 against leptospirosis [6]. Because we observed that sensitive animals mount a delayed inflammatory response, we hypothesized that an immune activator will be beneficial to control leptospirosis and tested the efficacy of LPS against lethal pathogenesis. Our results point to the possibility of treating leptospirosis by increasing inflammation level.

The early inflammatory response is important for controlling leptospirosis. Our results using LPS are consistent with the view that early innate immune response is crucial for inhibiting pathogenesis. Although the inflammation level was increased at 2-day p.i. in LPS-treated group, the leptospira burden was not changed until 4 day after infection. This suggests that the increased inflammation level may bolster the recognition of and response against leptospira, instead of killing the bacteria directly. Deletion of TLR2 and TLR4 makes the mice more sensitive to leptospira infection [6,12]. The induction of TLR2 is also delayed in hamsters compared with mice [6]. Thus, TLRs are key factors that differentiate between host susceptibility and protection.

In this study, we show that treatment with LPS promoted the gene expressions of TLR2, TLR4, NLRP3 and downstream cytokines, all of which would help control leptospirosis. In addition to innate immunity, acquired immunity, especially B cell response, facilitates the clearance of leptospira [11]. Our study also showed that the anti-leptospira IgG level was improved after treatment with LPS. Therefore treatment with LPS enhanced both innate and adaptive immunity against leptospira infection.

NO plays an important role in the systemic inflammatory response during leptospirosis [25]. For instance, massive pulmonary hemorrhage caused by leptospirosis can be successfully treated with nitric oxide inhalation and hemofiltration [26]. Furthermore, administration of 4-aminopyridine, an iNOS inhibitor, accelerated the mortality rate of hamsters and mice after leptospira infection [22]. In this study, we explore the role of iNOS on LPS-induced protective effect. The results showed that the expression of iNOS and the level of its product NO were elevated after treatment with LPS in kidney, liver and lung of hamsters. However, hamsters treated with 4-aminopyridine showed no significant difference compared with untreated group. Our results demonstrated that LPS-induced protective effect is iNOS-independent, although the detailed mechanism deserves further exploration.

In this study, we verify the efficacy of LPS against leptospirosis and point to the possibility of treating leptospirosis by increasing inflammation level. In the future, lots of effective immunoactivators will be studied for treating leptospirosis to determine which pathways are sufficient to evoke protective inflammation without causing pathological outcomes.
